# Supplementary material for: Rainforest transformation reallocates energy from green to brown food webs
Source: Nature. 2024 Feb 14;627(8002):116–22. doi: 10.1038/s41586-024-07083-y (PMC10917685; doi:10.1038/s41586-024-07083-y)
Supplement: Supplementary file 1 — Validation survey. [file 41586_2024_7083_MOESM1_ESM.pdf]

nature portfolio

https://doi.org/10.1038/s41586-024-07083-y

Supplementary information

Rainforest transformation reallocates energy from green to brown food webs

In the format provided by the
authors and unedited

Supplementary Notes – validation survey

Our validation survey showed that the total energy flux and biomass in rainforest was ca. 2.5 times lower than that in the main survey due to a lower abundance of earthworms, ants, and other dominant group (Extended Data Figs. 6 and 7). This may be a consequence of the drought that followed the El Niño events in 2015 the study region[1]. Despite this difference in the absolute values, the proportion of aboveground fluxes in the total energy flux was nearly the same (6.8% in the main and 8.1% in the validation survey; Extended Data Fig. 5). The proportion of birds in the total canopy energy flux in rainforest was also similar in both surveys (7.4% in the main and 6.6% in the validation survey). Earthworms were responsible for an average of 5.5% of the energy flux in rainforest and for 52-54% of the energy flux across plantations; both numbers were lower than in the main survey, but the strong differences among land-use types remained, and other energetically important groups were similar between the main and validation surveys (Extended Data Table 3). The belowground energy flux was manifold higher than the aboveground across land-use systems, and the total aboveground energy flux was reduced by -53 to -67% in both monoculture plantation types in comparison to rainforest (-75 to -79% in the main survey; Extended Data Fig. 5). We also confirmed a decline in the belowground predation/consumption ratio in monoculture plantations (-62 to -71%), and a moderate decline aboveground in oil palm (-8%), but not in rubber plantations (+8%). Similar changes in trophic functions were recorded in both surveys: total herbivory and fungivory were lower, while total bacterivory, soil feeding, and bacteria/fungi energy flux ratio were higher in monoculture plantations than in rainforest. However, in contrast to the main survey, our validation survey was unable to detect a significant reduction in the number of trophic interactions in plantation systems (except oil palm belowground; Extended Data Fig. 5). Overall, we have validated energetic dominance of the belowground over the aboveground energy channel, canopy arthropods over birds, energetic decline in canopies, and reallocation of energy to belowground food webs in plantations, and shifts in trophic functions, but were not able to validate food-web simplification across above- and belowground compartments.

1. Stiegler C. et al. ‘El Niño–Southern Oscillation (ENSO) event reduces CO2 uptake of an Indonesian oil palm plantation’, Biogeosciences 16, 2823–90 (2019).
