# Supplementary material for: Rainforest transformation reallocates energy from green to brown food webs
Source: Nature. 2024 Feb 14;627(8002):116–22. doi: 10.1038/s41586-024-07083-y (PMC10917685; doi:10.1038/s41586-024-07083-y)
Supplement: Supplementary file 2 — Reporting Summary [file 41586_2024_7083_MOESM2_ESM.pdf]

nature portfolio

Corresponding author(s): Anton Potapov

Last updated by author(s): Nov 28, 2023

Reporting Summary

Nature Portfolio wishes to improve the reproducibility of the work that we publish. This form provides structure for consistency and transparency in reporting. For further information on Nature Portfolio policies, see our Editorial Policies and the Editorial Policy Checklist.

Statistics

For all statistical analyses, confirm that the following items are present in the figure legend, table legend, main text, or Methods section.

n/a	Confirmed	
☐	☒	The exact sample size (*n*) for each experimental group/condition, given as a discrete number and unit of measurement
☐	☒	A statement on whether measurements were taken from distinct samples or whether the same sample was measured repeatedly
☐	☒	The statistical test(s) used AND whether they are one- or two-sided<br>*Only common tests should be described solely by name; describe more complex techniques in the Methods section.*
☐	☒	A description of all covariates tested
☐	☒	A description of any assumptions or corrections, such as tests of normality and adjustment for multiple comparisons
☐	☒	A full description of the statistical parameters including central tendency (e.g. means) or other basic estimates (e.g. regression coefficient) AND variation (e.g. standard deviation) or associated estimates of uncertainty (e.g. confidence intervals)
☐	☒	For null hypothesis testing, the test statistic (e.g. *F*, *t*, *r*) with confidence intervals, effect sizes, degrees of freedom and *P* value noted<br>*Give P values as exact values whenever suitable.*
☒	☐	For Bayesian analysis, information on the choice of priors and Markov chain Monte Carlo settings
☒	☐	For hierarchical and complex designs, identification of the appropriate level for tests and full reporting of outcomes
☒	☐	Estimates of effect sizes (e.g. Cohen's *d*, Pearson's *r*), indicating how they were calculated

Our web collection on statistics for biologists contains articles on many of the points above.

Software and code

Policy information about availability of computer code

Data collection	All data were collected from invertebrate samples and bird observations using manual counting and identification. No software was used during the data collection. BioSounds was used as a platform to get identification on the bird audio recordings (Darras, K., Pérez, N., Mauladi & Hanf-Dressler, T. BioSounds: an open-source, online platform for ecoacoustics. F1000Res. 9, 1224 (2020))
Data analysis	Data analysis was implemented in R v4.2.0 with R studio interface v1.4.1103 (RStudio, PBC). The following packages were used: lme4 v1.1-33, tidyverse v2.0.0, plyr v1.8.8, reshape v0.8.9, reshape2 v1.4.4, fluxweb v0.2.0, readxl v1.4.2, ggrepel v0.9.3, Food-web reconstruction code is openly available from Figshare (https://doi.org/10.6084/m9.figshare.24648438). Linear models are specified in the Extended Data Table 4.

For manuscripts utilizing custom algorithms or software that are central to the research but not yet described in published literature, software must be made available to editors and reviewers. We strongly encourage code deposition in a community repository (e.g. GitHub). See the Nature Portfolio guidelines for submitting code & software for further information.

Data

Policy information about availability of data

All manuscripts must include a data availability statement. This statement should provide the following information, where applicable:

- Accession codes, unique identifiers, or web links for publicly available datasets
- A description of any restrictions on data availability
- For clinical datasets or third party data, please ensure that the statement adheres to our policy

Raw data used in the analysis are available from Figshare: https://doi.org/10.6084/m9.figshare.24648438. The following datasets were used for bird identification and assignment of traits to invertebrates: Xeno-Canto online bird call database (http://xeno-canto.org/) and EltonTraits (Wilman, H. et al. EltonTraits 1.0: Species-level foraging attributes of the world's birds and mammals. Ecology 95, 2027–2027, 2014); Feeding habits review: Potapov, A. M. et al. Feeding habits and multifunctional classification of soil-associated consumers from protists to vertebrates. Biol. Rev. Camb. Philos. Soc. 97, 1057–1117 (2022); Stable isotope data: Zhou, Z., Krashevska, V., Widyastuti, R., Scheu, S. & Potapov, A. Tropical land use alters functional diversity of soil food webs and leads to monopolization of the detrital energy channel. Elife 11, (2022)

Human research participants

Policy information about studies involving human research participants and Sex and Gender in Research.

Reporting on sex and gender	N/A
Population characteristics	N/A
Recruitment	N/A
Ethics oversight	N/A

Note that full information on the approval of the study protocol must also be provided in the manuscript.

Field-specific reporting

Please select the one below that is the best fit for your research. If you are not sure, read the appropriate sections before making your selection.

☐ Life sciences ☐ Behavioural & social sciences ☒ Ecological, evolutionary & environmental sciences

For a reference copy of the document with all sections, see nature.com/documents/nr-reporting-summary-flat.pdf

Ecological, evolutionary & environmental sciences study design

All studies must disclose on these points even when the disclosure is negative.

Study description	The study explores differences between land-use systems (rainforest, jungle rubber, rubber, oil palm), and food-web compartments (aboveground, belowground), having a factorial design. In total, 32 sites were assessed, 8 in each system.
Research sample	The study analyses canopy and soil arthropods, birds, and earthworms in Jambi province, Sumatra, Indonesia.
Sampling strategy	The sampling was done on 8 independent sites (replicates) per system in two landscapes. No procedure was used to predetermine the sampling size.
Data collection	Authors of the paper applied a combination of collection methods to assess bird (point counts, sound recorders), canopy arthropod (fogging), soil arthropod and earthworm (heat extraction) communities.
Timing and spatial scale	The main sampling was done during May-October 2013 in two landscapes (around 'Harapan' and 'Bukit Duabelas' national parks) in Jambi province, Sumatra, Indonesia. Additional validation survey was done on 24 out of the 32 sampling sites in 2016-2017. Sampling sites were distributed over an area with a diameter of ca. 80 km.
Data exclusions	No data were excluded from the analysis
Reproducibility	We performed validation survey four years after the main survey on a subset of sites, which confirmed most of our conclusions. R code and statistical model specifications are openly available allowing to reproduce the data analysis.
Randomization	Sites, subplots within sites, and samples within subplots were randomly established taken. For more information see http://rstb.royalsocietypublishing.org/lookup/doi/10.1098/rstb.2015.0275
Blinding	This is an observational study dealing with animal biodiversity. Same people assessed different sites.

Did the study involve field work? ☒ Yes ☐ No

Field work, collection and transport

Field conditions	The climate is tropical humid with two peak rainy seasons around March and December, and a dryer period during July-August. Average annual temperature in the area is 26.7 ± 0.2°C, annual precipitation is 2235 ± 381 mm
Location	The study was carried out in Jambi province, Sumatra, Indonesia
Access & import/export	The permits for collection and export of the samples were granted by the Indonesian Ministry of Forestry (PHKA), Directorate General of Nature Resources and Ecosystem Conservation (KSDAE), and the Indonesian Institute of Sciences (LIPI). For soil invertebrates: collection permit no. S.07/KKH-2/2013 issued by PHKA, and export permit by LIPI (register file no. 24/SI/MZB/IV/2014) and PHKA (no. 125/KKH-5/TRP/2014). For canopy arthropods: collection Permit No. S.710/KKH-2/2013 issued by PHKA based on recommendation No. 2122/IPH.1/KS.02/X/2013 by LIPI, and export permit SK.61/KSDAE/SET/KSA.2/3/2019 issued by KSDAE based on LIPI recommendation B1885/IPH.1/KS.02.04/ VII/2017. For birds: field observations were carried out by Indonesian colleagues, so no research or collection permit was required. The research permit (number 211/SIP/FRP/SM/VI/2012) was recommended by LIPI and issued by PHKA. No bird collection or exporting was carried out. For the validation survey, the following research permits were used: canopy arthropods: 131/SIP/FRP/E5/Dit.KI/V/2017, birds: 386/SIP/FRP/E5/Dit.KI/XI/2016, soil invertebrates: 2841/IPH.1/KS.02.04/ X/2016 (collection permit), canopy height and tree stand properties: 42/EXT/SIP/FRP/E5/Dit.KI/VIII/2016 and 56/EXT/SIP/FRP/E5/ Dit.KI/IX/2017
Disturbance	Work on the study sites was implemented with care, to minimize disturbance. Whenever possible, manipulations with samples were done in a laboratory, outside the field sampling areas.

Reporting for specific materials, systems and methods

We require information from authors about some types of materials, experimental systems and methods used in many studies. Here, indicate whether each material, system or method listed is relevant to your study. If you are not sure if a list item applies to your research, read the appropriate section before selecting a response.

Materials & experimental systems

n/a	Involved in the study	
☒	☐	Antibodies
☒	☐	Eukaryotic cell lines
☒	☐	Palaeontology and archaeology
☐	☒	Animals and other organisms
☒	☐	Clinical data
☒	☐	Dual use research of concern

Methods

n/a	Involved in the study	
☒	☐	ChIP-seq
☒	☐	Flow cytometry
☒	☐	MRI-based neuroimaging

Animals and other research organisms

Policy information about studies involving animals; ARRIVE guidelines recommended for reporting animal research, and Sex and Gender in Research

Laboratory animals	Study did not involve laboratory animals
Wild animals	Only invertebrate animals (arthropods and earthworms) were collected and killed using ethanol during the study. This was necessary to assess biomass and community composition. We collected canopy arthropod communities using insecticide fogging, soil arthropod and earthworm communities using Kempson extractors and assessed bird communities using point counts and audio recorders. Presumably several hundreds of species (mainly unidentified) were collected without selection for age or strains.
Reporting on sex	Sex was not considered in the study
Field-collected samples	Collected soil samples were transported in the lab within 2-3 days for heat extraction. All invertebrates were stored in 70-80% ethanol solution. No field-collected environmental samples were used in this study.
Ethics oversight	No ethical approval was required. The study did not involve vertebrate animal capturing and killing

Note that full information on the approval of the study protocol must also be provided in the manuscript.
